# Agronomic monsoon onset definitions to support planting decisions for rainfed rice in Bangladesh

**DOI:** 10.1007/s10584-024-03736-z

**Published:** 2024-05-13

**Authors:** Eunjin Han, Carlo Montes, Sk. Ghulam Hussain, Timothy J. Krupnik

**Affiliations:** 1 Adaptive Cropping Systems Laboratory, USDA-Agricultural Research Service, Beltsville, MD 20705 USA; 2 International Maize and Wheat Improvement Center (CIMMYT), Texcoco, Mexico; 3 International Maize and Wheat Improvement Center (CIMMYT), Dhaka, Bangladesh

**Keywords:** Monsoon onset definition, South Asian monsoon, Bangladesh aman rice, Crop modeling

## Abstract

The usability gaps between climate information producers and users have always been an issue in climate services. This study aims to tackle the gap for rice farmers in Bangladesh by exploring the potential value of tailored agronomic monsoon onset definitions. Summer *aman* rice is primarily cultivated under rainfed conditions, and farmers rely largely on monsoon rainfall and its onset for crop establishment. However, farmers’ perception of the arrival of sufficient rains does not necessarily coincide with meteorological definitions of monsoon onset. Therefore, localized agronomic definitions of monsoon onset need to be developed and evaluated to advance in the targeted actionable climate forecast. We analyzed historical daily rainfall from four locations across a north-south gradient in Bangladesh and defined dynamic definitions of monsoon onset based on a set of local parameters. The agronomic onset definition was evaluated in terms of attainable yields simulated by a rice simulation model compared to results obtained using conventional meteorological onset parameters defined by the amount of rainfall received and static onset dates. Our results show that average simulated yields increase up to 7 – 9% and probabilities of getting lower yields are reduced when the year-to-year varying dynamic onset is used over the two drier locations under fully rainfed conditions. It is mainly due to earlier transplanting dates, avoiding the impact of drought experienced with early monsoon demise. However, no yield increases are observed over the two wetter locations. This study shows the potential benefits of generating “localized and translated” climate predictions.

**Supplementary Information:**

The online version contains supplementary material available at 10.1007/s10584-024-03736-z.

## Introduction

Adapting to climate change and variability is more challenging for developing countries because of the lack of capacity to deal with natural hazards from social, technical, political, and/or economic perspectives (Ayers et al. 2014). Along with the efforts to reduce society's vulnerability to climate change and related risks, climate services have gained prominence since 2012 with the establishment of the Global Framework for Climate Servies (GFCS) by the World Meteorological Organization (WMO) (Vaughan and Dessai 2014). Nonetheless, unsuccessful implementations of climate services have been partly attributed to a "lack of relevant and timely products and services offered by the scientific community"(Brasseur and Gallardo 2016).

This study aims to tackle the connection problem between climate information producers and users, and thus contribute to generating more usable climate services, in the present case of informing monsoon onset for rice farmers in Bangladesh. Information on the timing of the monsoon onset is crucial for livelihoods in Bangladesh, driving the decision-making of multiple stakeholders. The climate science community has made long-standing efforts to better understand the physical mechanisms of monsoon development and progression and, eventually, to advance in the predictions of monsoon timing in Bangladesh (Montes et al. 2021b). However, there have been historical 'usability gaps' between the knowledge producers (i.e., National Meteorological Services) and the actual users (i.e., extension workers or farmers in the case of agriculture) from the perspective of climate services (Lemos et al. 2012; Raaphorst et al. 2020). One of the distinctive examples can be found in the perception of the monsoon onset by agricultural workers, which varies by geographic location and does not necessarily agree with scientific definitions (Bremer et al. 2023; Stiller-Reeve et al. 2015). Ingesting end-users' perceptions in developing climate services is crucial to generate usable information beyond useful scientific knowledge.

In developing countries such as Bangladesh, smallholder farmers often have limited resources to cope with climate-related risks. However, management strategies such as shifting planting dates to minimize risks during critical growth stages can be a suitable approach that farmers are relatively able to act upon (Abdur Rashid Sarker et al. 2013; Alam et al. 2017; Sultan et al. 2005), and that has been shown to mitigate risks (Balwinder-Singh et al. 2019; Montes et al. 2022). In Bangladesh, *aman* rice farmers tend to prepare their nurseries around mid to late June and wait for the first significant rainfall to prepare their field before transplanting roughly 25-30 days old seedlings. A significant delay in transplanting due to the late arrival of monsoon rains can result in spikelet sterility and subsequent yield loss caused by water stress during panicle emergence and grain filling (Kabir et al. 2014; Nahar et al. 2009). Considering the heavy reliance on the monsoon seasonality by the rainfed rice farmers for nursery establishment and transplanting, the development of reliable forecasts of monsoon onset would be a crucial component for effective agricultural climate information services in Bangladesh (Kumar et al. 2020).

The development of skillful and usable forecasts of monsoon onset should be preceded by its appropriate definition to meet the users’ needs (Stiller-Reeve et al. 2015), for which multiple approaches have been proposed (e.g., Fitzpatrick et al. 2015). Examples include regional definitions based on the change in seasonal pattern of rainfall or convective activity (Ananthakrishnan and Soman 1988; Lau and Yang 1997; Wang and LinHo 2002), the shift in meridional wind direction (Holland 1986), or combined wind-convection criteria (An et al. 1998; Kueh et al. 2010; Matsumoto 1997; Wang and Wu 1997). Local definitions in which the meteorological information from a single point is processed to define the onset of the monsoon have also been developed (Marteau et al. 2009; Moron and Robertson 2014). In Bangladesh, important differences in monsoon onset dates were described by Stiller-Reeve et al. (2015), who evaluated monsoon progressions based on 11 different definitions from the literature, showing similar patterns of monsoon progression but some exceptionally early onsets in some cases. Despite the scientific consensus on the monsoon progression, local agricultural communities' perceptions of the monsoon onset, definition, and interannual variability were different by geographic location.

Keeping agricultural applications in mind, local agronomic definitions of monsoon onset have been applied over monsoon areas of Africa and South Asia (Fitzpatrick et al. 2016; Marteau et al. 2009; Moron and Robertson 2014). This approach is based on determining threshold parameters describing rainfall features such as the initial wet spell and post-onset dry spell in terms of duration and amount of rainfall based on long-term daily statistics, but also variables describing local water balance. These threshold parameters are descriptors of soil conditions for crop establishment and growth, helping to avoid false onsets that can result in early crop failure. These definitions, which are more suitable for agricultural applications, tend to differ from those used by national meteorological services and climatologists, often based on meteorological variables only. Moreover, detailed analysis using local agronomic onset definitions have been carried out only in a few countries in Africa (Dodd and Jolliffe 2001; Marteau et al. 2009; Marteau et al. 2011) and in India (Moron and Robertson 2014). In a large-scale analysis over the Indian subcontinent, Fitzpatrick et al. (2016) analyzed the spatial coherence of the agronomic monsoon onset using satellite-derived precipitation data and the definition of Marteau et al. (2009), though Bangladesh was not considered. To our knowledge, no efforts have however been made to determine agronomically relevant onset criteria in Bangladesh. In addition, no previous studies have evaluated the utility of local agronomic definitions against those more commonly employed by meteorologists that are based mainly on cumulative rainfall or other meteorological variables (e.g., wind direction, water vapor transport). Furthermore, potential benefits of the agronomic definitions to agricultural decision makers have not been quantified in terms of crop yields.

In response to these challenges, we analyzed historical daily rainfall data from four regional weather stations located across a north-south gradient in Bangladesh over locations where rainfed aman rice production predominates. We calculated threshold parameters and long-term monsoon onset dates using the agronomic onset definitions. Then, we conducted an ex-ante assessment of the utility of the agronomic definitions through comparison with the performance of other three reference onset definitions in terms of attainable yields of rainfed rice using the Decision Support System for Agrotechnology Transfer (DSSAT)- Crop Environment Resource Synthesis (CERES) - rice model and 36 years of historical weather observations. Specifically, we examined how agronomic definitions of monsoon onset could potentially help rice farmers make improved decisions on the timing of nursery preparation and transplanting. The study provides an example of how demand-driven climate information (i.e., monsoon onset for farmers’ planting decision support) could be customized to better contextualize climate services (e.g., seasonal or sub-seasonal forecasts).

## Meteorological data

**Fig. 1:**
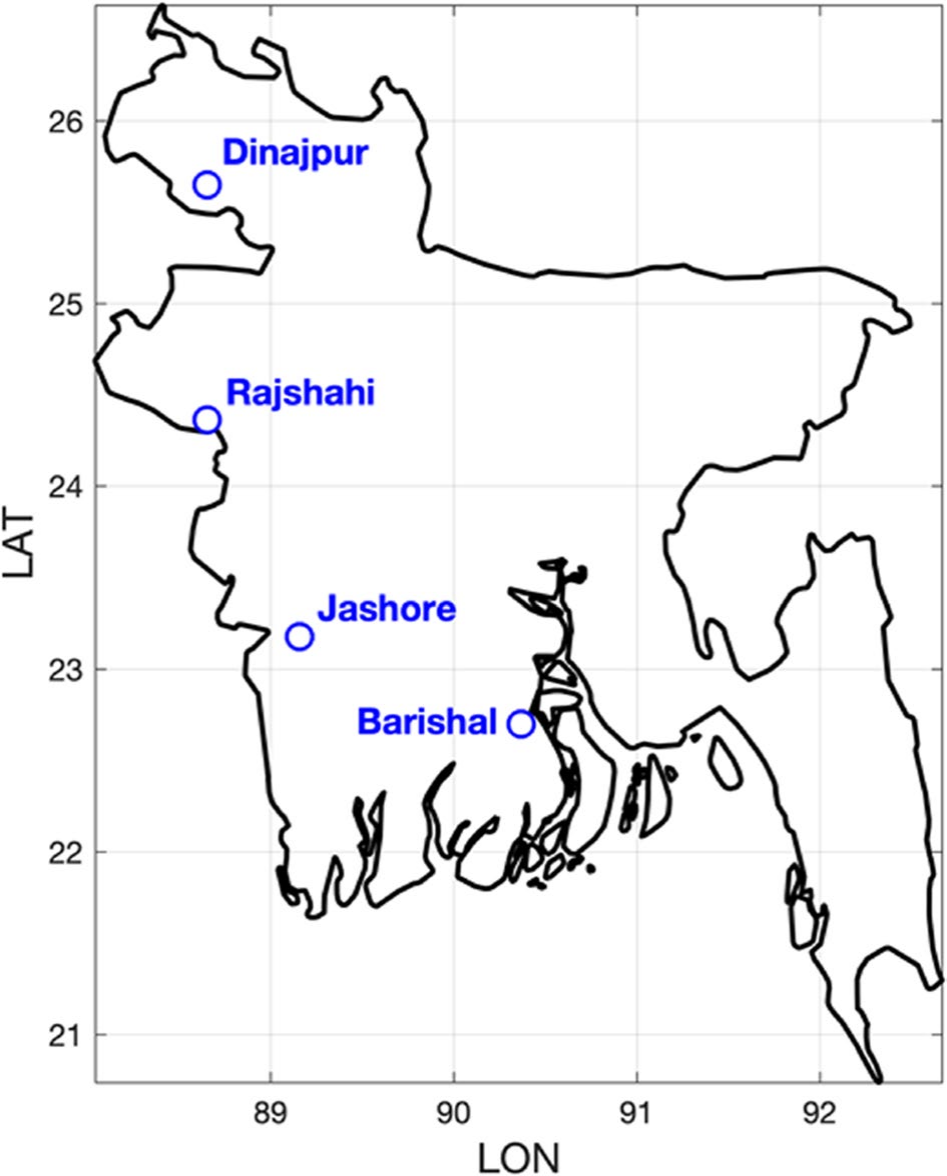
Map of Bangladesh showing the location of the four weather stations used

The study was carried out using data from four locations in Bangladesh (Fig. 1). They were selected to represent major *aman* production areas in the Western part of the country by excluding high monsoon rain regions (Sylhet and Chittagong) and areas non-suitable for *aman* rice (Al Mamun et al. 2021; Hussain et al. 2014; Montes et al. 2021a). The four weather stations were selected according to the availability of data necessary for both the implementation of the monsoon onset method and crop modeling. Despite the limited number of locations, they can represent an early to late gradient of monsoon onset (Montes et al. 2021b). Daily rainfall, minimum and maximum air temperature, sunshine hours, relative humidity and wind speed data for the period 1981-2017 were provided by the Bangladesh Meteorological Department (BMD). Given that solar radiation is not measured by BMD, an important variable for crop modeling, the empirical regression-based Angström-Black linear model (Ampratwum and Dorvlo 1999) was used to estimate solar radiation from sunshine hours.

**Fig. 2:**
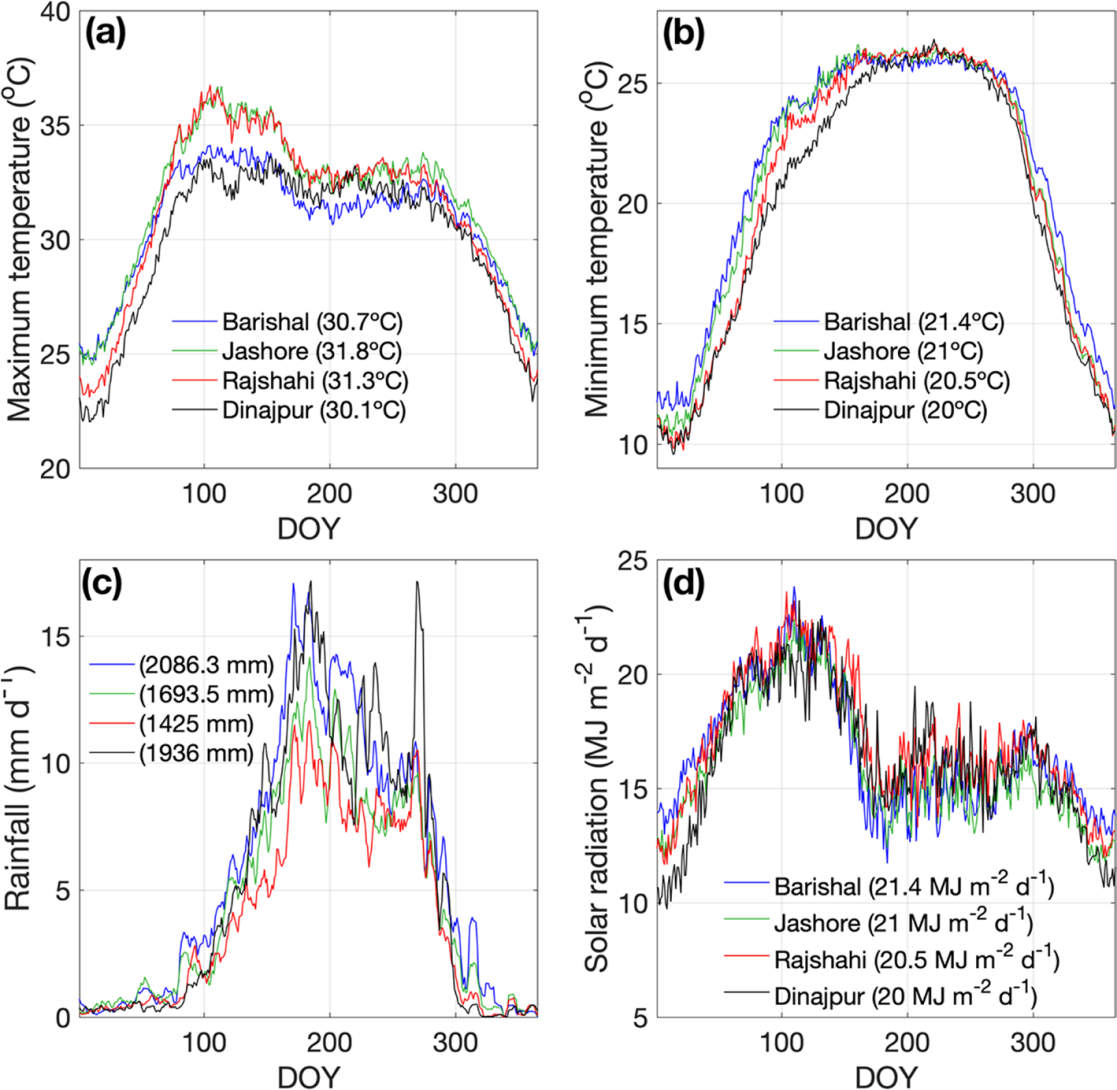
Mean annual cycle (1981-2017) of (**a**) daily maximum temperature, (**b**) daily minimum temperature, (**c**) 7-day rainfall moving average, and (**d**) daily solar incoming radiation for the four locations in Bangladesh. DOY indicates ‘day of year’. The values in parentheses correspond to the long-term annual averages

Long-term (1981-2017) climatology and mean annual cycle of air temperature, precipitation and solar radiation are displayed in Fig. 2. Similarly, interannual variability is presented in Figs. S1-S4. Climatological rainfall (Fig. 2c) shows that there are significant differences between locations ranging from 1,425 to 2,086 mm year^-1^, accounting for a Southwest-Northeast gradient in annual rainfall (Shahid 2009). The mean annual cycle of rainfall shows a strong increase of precipitation towards the summer from values close to zero in winter. Barishal and Dinajpur present lower maximum temperatures during the monsoon onset transitions, associated with the higher precipitation of these two stations.

## Methods

### Defining a tailored agronomic monsoon onset date

Several local and regional definitions of monsoon onset date have been developed for different monsoon regions (e.g. Matsumoto 1997; Stiller-Reeve et al. 2014; Zhang et al. 2002). In this work, we followed the agriculturally specific ‘agronomic’ monsoon onset approach proposed by Marteau et al. (2009) and Moron and Robertson (2014) to define the local monsoon onset date. Moron and Robertson (2014) state that the date of the agronomic onset should be calculated reflecting locally-varying precipitation parameters (e.g. length of wet or dry spells) to consider local climate conditions and soil water balance. As such, the following four parameters characterizing the daily rainfall evolution have to be defined at the local-scale: (1) length of the initial wet spell (LWS), (2) the amount of rainfall received during the initial wet spell (AWS), (3) length of post-onset dry spell (LDS) to avoid false onsets related to pre-monsoon rainfall, and (4) the maximum amount of rainfall received during the post-onset dry spell (ADS). Previous studies in Bangladesh have defined monsoon onset and withdrawal climatological dates from the first half of June and the first half of October (Ahmed and Karmakar 1993) or early June (between May 31^st^ and June 9^th^) and early October (between October 3^rd^ and 12^th^) (Hoque et al. 2011). Based on this information, we applied the period between May 15^th^ and October 31^st^ to compute the above presented parameters for the four selected weather stations (Fig. 1) and presented them in Table 1.

**Table 1:** Locations and parameters for monsoon onset date calculation

Location	Latitude (°)	Longitude (°)	ET_0_ (mm day^-1^)	LWS (days)	AWS (mm)	LDS (days)	ADS (mm)
Barishal	22.70	90.37	3	4	55	5	19
Jashore	23.18	89.16	3	3	27	5	17
Rajshahi	24.36	88.65	4	3	31	6	11
Dinajpur	25.65	88.67	4	3	34	6	12

*LWS* length of the initial wet spell, *AWS* amount of rainfall during the initial wet spell, *LDS* length of the post-onset dry spell, *ADS* amount of rainfall of the post-onset dry spell

The LWS is the minimum number of consecutive rainy days (rainfall ≥ 1 mm, number of days ≥ 1) and was calculated by taking the average length of wet spells between May 15^th^ and October 31^st^. BMD data indicate that wet spells for the four locations range from two to three days, with a standard deviation of one day over the four locations. Consequently, an LWS equal to the average length of wet spells plus 1 standard deviation was considered as appropriate to define the initial wet spell (Table 1).

Moron and Robertson (2014) argued that the AWS should be set following an agricultural water balance criterion considering the amount of water needed for germination of seeds. The mean reference evapotranspiration (ET_0_), calculated using the FAO-56 method (Allen et al. 1998), during the end of the dry season period from March 1^st^ and May 31^st^ averages from 3 to 4 mm d^-1^ (Table 1). The amount of rainfall during the initial wet spell (AWS) was calculated as the average total rainfall accumulated during wet spells of all years (1981-2017) lasting at least the corresponding LWS (Table 1) and whose daily rainfall equals or exceeds the average evaporative demand (ET_0_), values that would represent a minimum amount to wet the upper soil layers. For instance, in the case of Barisal, at least 12 mm of rainfall (3 mm day^-1^ of ET_0_ multiplied by 4 days of LWS) is required to wet the upper soils for seed germination and thus 55 mm of AWS is adequate for sowing, and as a criteria for agronomic onset definition. (Allen et al. 1998)The average LDS, defined to avoid false onsets, during May 15^th^ and October 31^st^, was obtained by calculating the average length of dry spells in which the number of consecutive days with rainfall ≤ 1 mm could be summed. Our calculations show that the length of dry spells ranges from four to five days, with a standard deviation of one day. The latter suggests that a post-onset dry spell of 5-6 days (LDS, Table 1) is a reasonable parameter to avoid false monsoon onsets. Finally, the ADS was obtained by calculating the average ET_0_ during the period May 15^th^ and October 31^st^. Our results show that ET_0_ ranges from 3 to 4 mm d^-1^, which would evaporate an amount of water of average 11 to 19 mm during the LDS (ADS, Table 1). ADS was calculated as the product between the post-onset length of the dry spell (LDS) and the average ET_0_, which gives the theoretical amount of rainfall (mm) necessary to fill the upper soil layers (days × mm d^-1^).

Thus, for each station, monsoon onset date is defined as the first wet day from May 15^th^ of the first wet spell of LWS days receiving at least the amount of rainfall AWS, without being followed by dry spell of LDS days receiving less rainfall than ADS in the subsequent 20 days from the onset (Table 1).

### DSSAT CERES-Rice simulation

The DSSAT is a software package that consists of multiple modules to simulate crop growth and yields and the interactions between crops, soil and atmospheric environment at a spatially uniform field (Hoogenboom et al. 2019; Jones et al. 2003). The CERES-Rice model was developed in 1990s (Buresh et al. 1991; Ritchie et al. 1998; Ritchie et al. 1987; Sing et al. 1990; Singh et al. 1993) and later embedded to the DSSAT package (Jones et al. 2003). The input data to run the CERES-Rice model include daily weather data (i.e., maximum and minimum air temperature, solar radiation and rainfall), soil physical and chemical properties, genetic coefficients of a cultivar and management practices (i.e., planting date, irrigation, fertilizer application etc.). Rice phenology and its rate of development are simulated using accumulated thermal time, called growing degree days, which is calculated from daily air temperature (Jones et al. 2003). The growing degree days required for transition to each growth stage are determined by user-defined genetic coefficients (Jones et al. 2003). Detailed soil properties of each layer are needed to compute water, carbon and nitrogen balances, soil temperature and soil-plant-atmosphere interactions. Total crop biomass is mainly determined by intercepted photosynthetically active radiation and reduced by several stress factors, such as extreme temperature or limited water or nitrogen availability (Ritchie et al. 1998).

The DSSAT CERES-Rice model has been widely evaluated in several countries with monsoonal rainfall regime including Thailand (Cheyglinted et al. 2001), China (Zhang et al. 2017) and India (Sarkar and Kar 2006; Singh et al. 2007). In Bangladesh, The CERES-Rice model has been used to assess rice productivity under changing climate and the impact of transplanting dates and soil water stress on potential yields (Basak et al. 2013; Hussain 2011; Hussain et al. 2014; Mahmood et al. 2004; Mahmood et al. 2003; Maniruzzaman et al. 2017; Yu et al. 2010).

In this study, we used calibrated parameters from Hussain et al. (2014) for genetic coefficients (Table S1) of the high-yielding medium-duration *indica* rice variety BR 11, the most commonly cultivated *aman* season variety in Bangladesh (Mahmood et al. 2003; Mahmood et al. 2004; Shelley et al. 2016). Hussain et al. (2014) showed the performance of model-predicted yields at district and division levels. For instance, the coefficient of determination between simulated and observed yields for a target year (2013) was 0.603 and 0.497 at the district and division level, respectively. Information on typical planting and management, and fertilizer application parameters were taken from Hussain et al. (2014) and used to reflect common practices after consulting with local agricultural experts in Bangladesh. Each study location has different soil types including *Barishal, Gangni, Ishurdi,* and *Ruhea* series for Barishal, Jashore, Rajshahi and Dinajpur, respectively (Hussain et al. 2014). Soil properties and profiles are presented in Table S2. We also adopted management parameters for rainfed *aman* rice from Hussain et al. (2014), namely plant population at emergence, row spacing, and transplanting depth were set as 25 plants m^-2^, 20 cm and 3 cm, respectively. Similarly, N as urea, P as triple super phosphate and K as potassium chloride were considered as sources of inorganic fertilizers. In the model, urea was applied in three equal splits at final land preparation and at 25 and 60 days after transplanting with same amount of 28 N kg ha^-1^ application^-1^. Phosphate and potassium fertilizers were applied 20 P kg ha^-1^ and 35 K kg ha^-1^, respectively, at the time of land preparation. The N fertilizer applications are similar to the optimum management practice recommended by the Bangladesh Rice Research Institute. Transplanting age and date varied by year when dynamic onset definitions were applied.

Our modeling experiments assumed fully rainfed conditions to focus on the impacts of inter-annual weather variability not mediated by irrigation. Bund height was set up as 15 cm. Transplanting date was determined when threshold ponding water depth (30 mm) was reached by rainfall. Puddling was set a day before transplanting. Percolation rates were set at 2 mm day^-1^. An example experiment file (RIX) we used in this study can be found in Table S4.

### *Ex-ante* crop simulation experiments

We investigated the differences associated with the use of two static and two dynamic (i.e., variable year-to-year) criteria for nursery establishment and transplanting dates, and their effects on rice yields under fully rainfed conditions, in order to evaluate the utility of agronomic onset definition (Section 3.1). The main assumption is that farmers establish their nursery when the monsoon starts and therefore, the way in which farmers identify monsoon onset determines nursery establishment dates. First, a traditional practice is to consider a specific date as the onset of the rainy season (i.e., date for nursery establishment), typically the first day of the Bengali month of *Ashar*, equivalent to June 15^th^ in the western calendar (Stiller-Reeve et al. 2015). In addition, farmers are encouraged by extension services to prepare their nurseries on June 15^th^ and then wait for the first rain for transplanting (Chowdhury and Hassan 2013). Consequently, in our simulation experiments farmers make their nursery on June 15^th^ every year and wait for transplanting until the seedlings are at least 21 days old and their rice field has threshold ponding depth (30 mm) after a significant rainfall event. Our seedling age criteria followed those proposed by Balwinder-Singh et al. (2019), a few days earlier than the 25-35 day-old range identified by Chowdhury and Hassan (2013). We adjusted ponding depth criteria to 30 mm, which is lower than the 50 mm used by Balwinder-Singh et al. (2019) for north India, in order to reflect local practices (cf. Rashid et al. 2018), and to avoid unrealistically late transplanting dates. This scenario is called Static 1 hereafter.

Secondly, based on a previous field survey about farmers’ own perceptions of the conditions associated with the monsoon onset across Bangladesh conducted by Stiller-Reeve et al. (2015), we assumed that *aman* nurseries were prepared on the *normal* onset dates reported by agricultural workers. These dates are May 31^st^, June 5^th^, and June 10^th^ for Barishal, Jashore and Rajshahi respectively. In the case of Barishal, the *normal* onset date is May 31^st^ which was taken from survey results in Chandpur, approximately 68 km from Barishal (refer to Fig. 4 in Stiller-Reeve et al. 2015). Similar to the previous scenario (Static 1), static dates for monsoon onset and nursery establishment are used but, in this case, they are location-specific with 5 – 10 days of difference between locations. This scenario is called Static 2 hereafter. The above-mentioned transplanting rule (at least 21 days old seedling and 30 mm of ponding depth) was subsequently applied to the second scenario.

As a third scenario, we examined the option that farmers establish nurseries considering a fixed accumulated rainfall amount. For this dynamic criterion, we followed the approach of Balwinder-Singh et al. (2019), where a nursery is prepared when accumulated rainfall during three consecutive days within the nursery sowing window (from May 15 to August 15) is equal to or greater than 50 mm. The above same rule is applied for transplanting. That is, as soon as three-day cumulative rainfall reaches the 50mm threshold, farmers prepare a nursery and wait until they have enough rainfall to fill the 30mm ponding depth and at least 21 days old seedlings. This scenario is called Dynamic 1 hereafter. Note that the Dynamic 1 method considers only three days of cumulative rainfall amount, not the following dry spells, and thus has a risk of giving rise to ‘false starts’ of the monsoon during the pre-monsoon season. A post-onset dry spell longer than 7 days could be damaging during the early vegetative stage (Moron and Robertson 2014).

Lastly, we applied the agronomic monsoon onset definition described in Section 3.1 as a second dynamic criterion for nursery establishment dates. Similar to Dynamic 1, this approach also considers inter-annual variability of the onset of the rainy season, but in this scenario, crop establishment is carried out following the agronomic monsoon onset estimation as a location-specific function of meteorological variables, which allows, for example, minimizing the occurrence of false onsets. This scenario is called Dynamic 2 hereafter. Again, transplanting follows the same rule (i.e., at least 21 days old seedling and 30 mm of ponding depth) as the previous cases. Note that when the above-mentioned transplanting rule resulted in transplanting dates later than September 7^th^ (DOY=207), we assumed no transplanting and thus no crop yield available for analysis.

The effect of different onset definitions was analyzed by several methods, including visualization, year-to-year comparison of simulated yields, comparison of full distributions of simulated yields using boxplots, and statistical analysis for testing mean and different percentiles. The equal mean hypothesis was tested using *t*-tests. We used the pairwise permutation test to compare different percentiles (10th, 25th, 50th, 75th, and 90th) of two distributions, using the *percentileTest* function in the *rcompanion* package in R.

## Results

### Monsoon onset dates from dynamic definitions

**Fig. 3:**
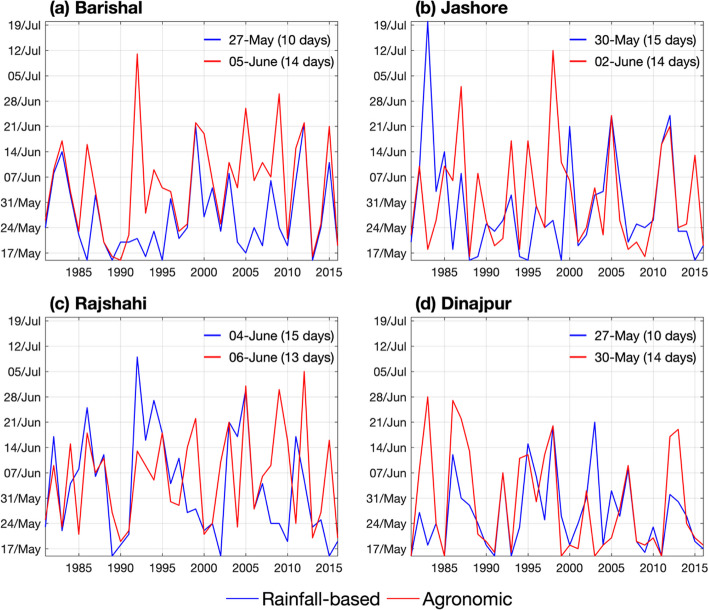
Time series (1981-2016) of monsoon onset dates obtained from the rainfall-based method (Dynamic 1) and the agronomic onset definition (Dynamic 2) for the four locations in Bangladesh. On each panel, the average date and standard deviation (in parentheses) are displayed

On average, onset dates of the Dynamic 1 range from May 27^th^ in Barishal and Dinajpur, to June 4^th^ in Rajshahi (Fig. 3). The dates obtained by Dynamic 2 vary from May 30^th^ in Dinajpur to June 6^th^ in Rajshahi. These mean values and range of interannual variability agree with previous studies (Ahmed and Karmakar 1993; Montes et al. 2021b; Stiller-Reeve et al. 2014), as well as the spatial pattern of later onset dates over Rajshahi and earlier over Barishal and Dinajpur. The time series of onset dates also indicate a relatively high interannual variability obtained by both methods, with a standard deviation ranging from 10 to 15 days. Particularly, relatively drier areas (Jashore and Rajshahi) show higher interannual variability than the wetter areas (Barishal and Dinajpur). Although the dates obtained with the two dynamic methods are similar, it is clear that the agronomic definition provides later onset dates, with an average difference ranging from two (Rajshahi) to nine days (Barishal).

### Ex-ante crop simulations using dynamic and static monsoon onset dates

**Fig. 4:**
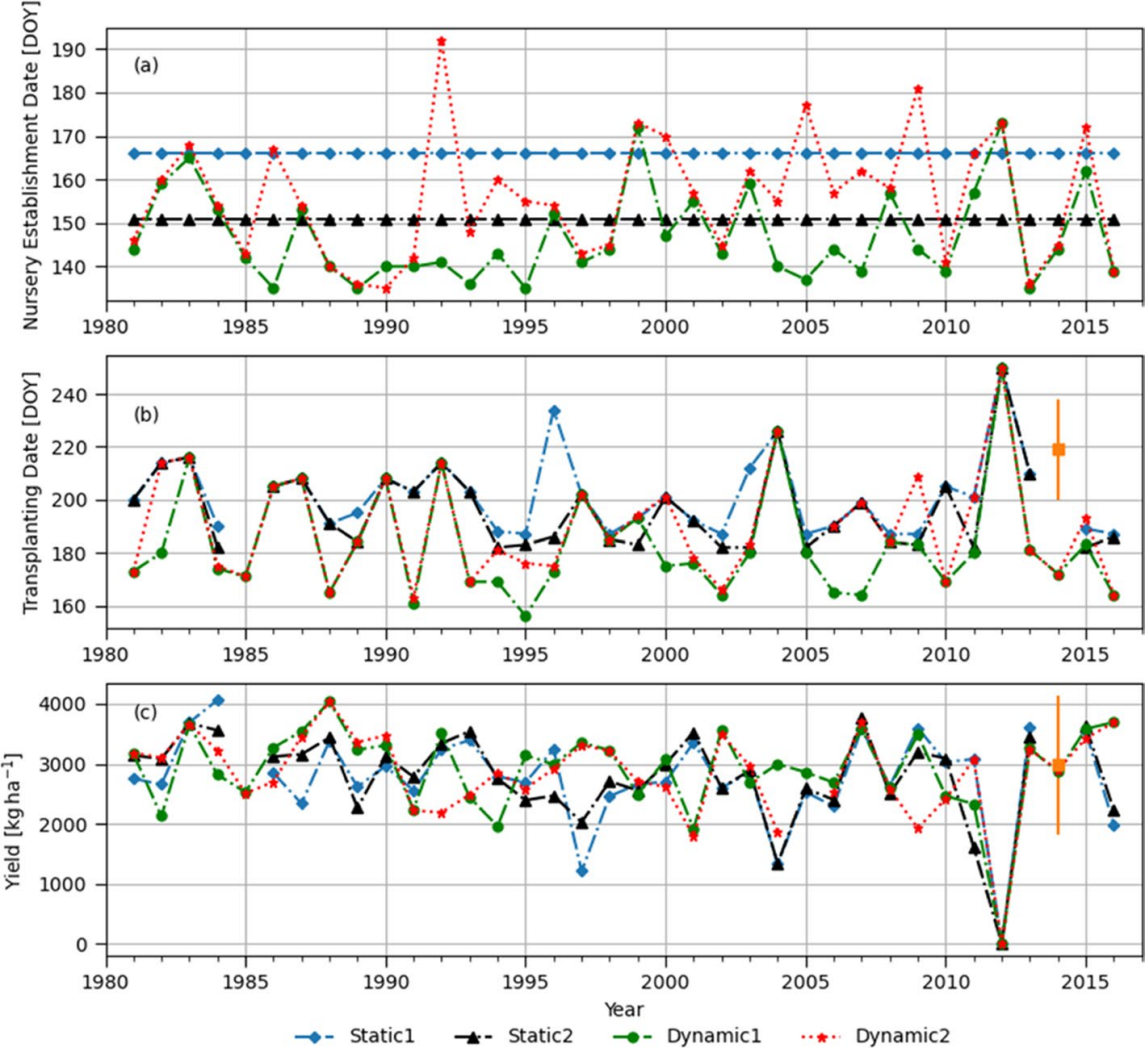
(**a**) Dates of nursery establishment corresponding to different monsoon onset definitions, (**b**) transplanting dates shown by day of year (DOY), and (**c**) simulated yields in Barishal. Static 1 and Static 2 represents nursery established on June 15^th^ and May 31^st^ every year. Dynamic 1 represents nursery established when cumulative rainfall of three consecutive days is greater than 50 mm while Dynamic 2 is based on the agronomic definition described in Section 3.3.1. Squared marks and error bars represent the mean and ± standard deviation of plot-level BIHS survey data

Model simulated transplanting dates and rice yields were compared with the field data taken from Bangladesh Integrated Household Survey (BIHS) (*International Food Policy Research Institute* 2016; Sapkota et al. 2021). We used the field data collected in 2015 throughout the four divisions where our target weather stations are located (Fig. S5). The field survey data have a wide range of variability in transplanting dates (i.e., 19 days of standard deviation) and yields (i.e., greater than 1000 kg ha of standard deviation), as shown in Table S3. That could be explained by numerous factors affecting transplanting and crop yields, such as preceding crops, labor availability, soil types, and farmers’ skills and financial capacity for production cost. Model estimated transplanting dates were earlier than the field survey results, which might reflect farmers’ risk aversiveness (i.e., delayed planting to avoid false onsets or prolonged dry spells during the pre-monsoon period) or late harvesting of preceding crops. Simulated rice yields in 2014 were close to the averages of the surveyed yield data or within the range of standard deviations for Barisal and Jashore but not for Dinajpur (Fig. 4, 6, and 8). Note that Dinajur had a minimal amount of rainfed data, and large numbers of farmers in Dinajur seemed to rely heavily on irrigation compared to other locations (Table S3). This comparison with the field survey data for a specific year (2014) confirmed that model simulation results are reasonable to use for the objective of the present study despite some deviations from the field data.

**Fig. 5:**
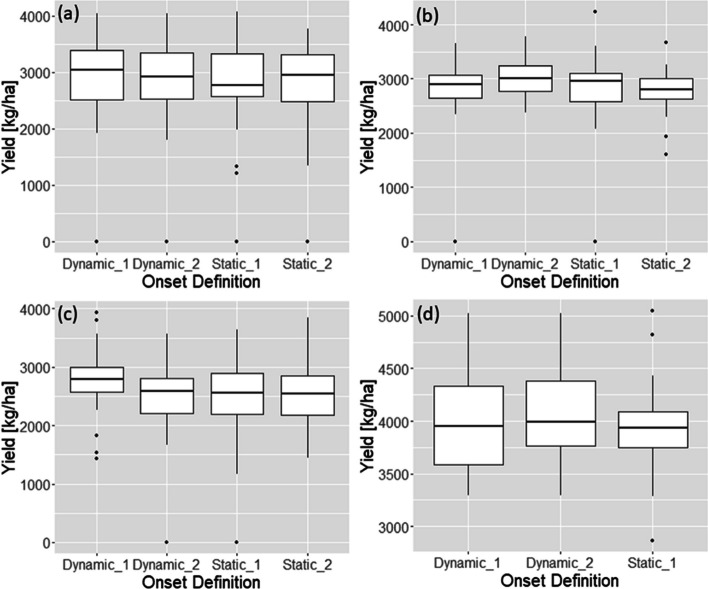
Boxplots of simulated yields for different monsoon onset definitions: (**a**) Barishal, (**b**) Jashore, (**c**) Rajshahi, and (**d**) Dinajpur

It should be emphasized that this study aims to evaluate “relative” values of four different onset definitions through controlled experiments (i.e., crop model simulations), not reflecting all heterogeneities in the actual farming communities but focusing on inter-annual weather variabilities of the real world. Thus, the utility of different onset definitions should be evaluated in terms of the whole distribution of 36 years of simulated yields (i.e., covering years with extremely early monsoon onset, normal years, and years with late-onset data), not individual years. Therefore, the nonparametric permutation statistical test results in Table 3 and box plots in Fig. 5 deliver the core results of the study. Nonetheless, the following sub-sections describe how different onset definitions affect transplanting dates and simulated rice yields differently for individual sites or years and explain some mechanisms contributing to the differences.

#### Barishal

The interannual variability of monsoon onset dates in Barishal obtained using the Dynamic 2 method are within the range of locally perceived onset dates reported by Stiller-Reeve et al. (2015), with the earliest falling on May 11^th^ (DOY=131) and the latest onset on June 30^th^ (DOY=181) in Chandpur (near Barishal) (Fig. 4a). Both dynamic onset definitions resulted in relatively earlier onsets than the two static onset dates (Fig. 4a). Particularly, the Dynamic 1 allowed 67% of onsets to occur in May and earlier than the Dynamic 2 in several years. It is mainly because it did not take into account for possible post-onset dry spells after the initial wet spell. Compared to the static onset assumption as a trigger for seedbed establishment (i.e., nurseries are prepared on June 15^th^, DOY=166, as generally recommended by national research and extension services), the dynamic onset definitions result in relatively earlier transplanting dates starting from early June (Fig. 4b). A delayed transplanting beyond the reasonable cropping time window (later than August 15^th^, DOY=227) resulted in crop failure in 2012 for all onset definitions. The four onset definitions did not result in statistically significant difference in simulated yields (Table 2 and 3, and Fig. 5) in spite of the difference in nursery establishment and transplanting dates.

**Table 2:** Summary statistics of simulated yields for the four monsoon onset definitions.

Location	Onset	N	Simulated Yield [Kg ha^-1^ ]						
			Mean	SD	Min	25^th^ percentile	Median	75^th^ percentile	Max
Barishal	D1	36	2909	728	0	2518	3034	3385	4037
	D2	35	2840	744	0	2520	2919	3338	4037
	S1	34	2773	787	0	2569	2772	3327	4063
	S2	34	2793	764	0	2479	2945	3306	3768
Jashore	D1	35	2820	580	0	2653	2892	3065	3646
	D2	35	3011	336	2370	2774	3000	3252	3781
	S1	34	2816	653	0	2578	2953	3107	4230
	S2	35	2803	383	1599	2627	2808	3011	3674
Rajshahi	D1	33	2764	546	1434	2580	2788	3000	3927
	D2	31	2523	643	0	2201	2586	2809	3572
	S1	31	2459	733	0	2193	2551	2891	3634
	S2	32	2542	636	1441	2174	2543	2853	3852
Dinajpur	D1	34	3998	445	3295	3582	3955	4333	5021
	D2	34	4054	415	3295	3768	3995	4383	5021
	S1	32	3941	417	2863	3745	3934	4087	5045

The table did not included years when transplanting was not found, but include zero yield due to unsuccessful grain filling. N indicated the number of simulated yields used for the statistics. SD represents the standard deviation. D1, D2, S1 and S2 represent Dynamic 1, Dynamic 2, Static 1 and Static 2 respectively. Refer to Fig. 4 for the definition of each onset method

**Table 3:** Statistical significance (*P*-values) of permutation tests comparing means and representative percentiles between two simulated yield distributions by different onset definitions

(a) Barishal	10^th^ percentile	25^th^ percentile	50^th^ percentile	75^th^ percentile	Mean
D1 – D2	0.751	0.992	0.701	0.863	0.696
D1 – S1	0.851	0.806	0.255	0.776	0.428
D1 – S2	0.818	0.818	0.790	0.785	0.486
D2 – S1	0.900	0.733	0.537	0.967	0.723
D2 – S2	0.930	0.773	0.954	0.927	0.794
S1 – S2	0.988	0.587	0.478	0.892	0.915
(b) Jashore	10^th^ percentile	25^th^ percentile	50^th^ percentile	75^th^ percentile	Mean
D1 – D2	0.231	0.192	0.210	0.128	0.095
D1 – S1	0.453	0.350	0.654	0.815	0.966
D1 – S2	0.596	0.745	0.477	0.697	0.887
D2 – S1	**0.038**	0.137	0.772	0.165	0.102
D2 – S2	0.108	0.177	**0.047**	**0.027**	**0.018**
S1 – S2	0.863	0.522	0.177	0.344	0.911
(c) Rajshahi	10^th^ percentile	25^th^ percentile	50^th^ percentile	75^th^ percentile	Mean
D1 – D2	0.518	***0.056***	**0.042**	0.150	0.169
D1 – S1	0.291	**0.028**	***0.051***	0.304	***0.061***
D1 – S2	0.124	0.045	**0.035**	0.565	0.132
D2 – S1	0.498	0.929	0.641	0.681	0.489
D2 – S2	0.139	0.875	0.786	0.843	0.884
S1 – S2	0.728	0.961	0.957	0.849	0.551
(d) Dinajpur	10^th^ percentile	25^th^ percentile	50^th^ percentile	75^th^ percentile	Mean
D1 – D2	0.348	0.187	0.754	0.717	0.593
D1 – S1	0.931	0.342	0.821	0.156	0.532
D2 – S1	0.692	0.834	0.619	0.089	0.222

D1, D2, S1 and S2 represent Dynamic 1, Dynamic 2, Static 1 and Static 2 respectively. Refer to Fig. 4 for the definition of each onset method. *p*-values below the 5%/10% level of significance indicates rejection of the null hypothesis as indicated in bold and underbar/bold and italic

#### Jashore

**Fig. 6:**
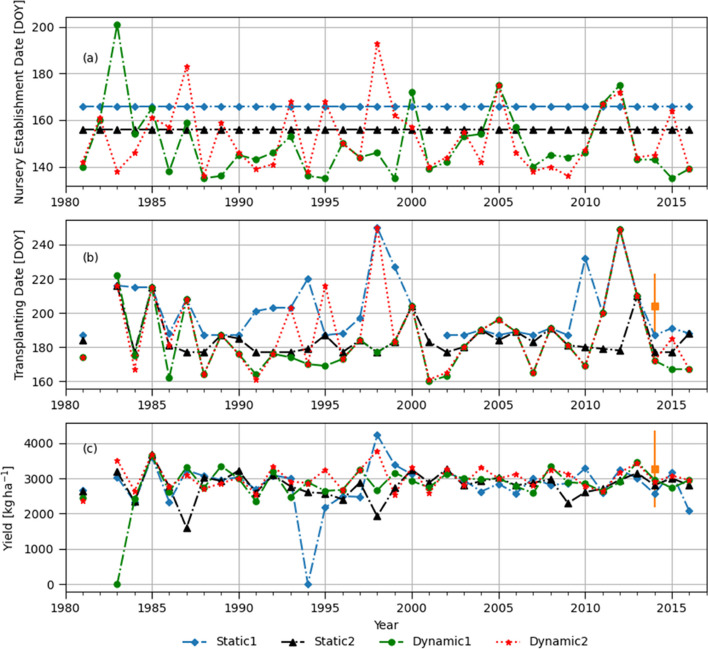
(**a**) Dates of nursery establishment corresponding to different monsoon onset definitions, (**b**) transplanting dates shown by day of year (DOY), and (**c**) simulated yields in Jashore. Refer to Fig. 4 for the definitions of Static 1, Dynamic 1 and Dynamic 2. Static 2 represents nursery established on June 5^th^ every year. Squared marks and error bars represent the mean and ± standard deviation of plot-level BIHS survey data

The locally perceived onset dates in Jashore from Stiller-Reeve et al. (2015) range from May 16^th^ (DOY=136) to July 15^th^ (DOY=196), being the latest date much later than the other stations in Bangladesh (e.g., June 30^th^ in both Rajshahi and Chanddpur, presented later). Both Dynamic 1 and Dynamic 2 definitions are able to capture the high interannual variations over Jashore (Fig. 6a). Particularly, the very late onset obtained in 1998 (DOY=193) using the Dynamic 2 definition reflects relatively low rainfall in June and July of 1998 (monthly rainfalls were 163/181 mm in June/July in 1998, much lower than long-term average of 286/320 mm). The exceptionally late onset date of July 20^th^ (DOY=201) in Fig. 6a identified by the Dynamic 1 in 1983 was due to the specific rainfall threshold values used (i.e., 50 mm over three consecutive days). There were wet spells before DOY=201, which accumulate 45 mm for two consecutive days and 80 mm for six consecutive days not meeting the rule of 50 mm for three days. This result shows a weakness of specific parameter-based onset definitions (i.e., too sensitive to fixed criteria).

In general, dynamic onset definitions provide earlier nursery establishment dates than the static definitions (Fig. 6a). Unlike the results for Barishal (Fig. 4a), there are a greater number of years (i.e., 10 years) when the Dynamic 2 resulted in earlier onsets than the Dynamic 1. This is because in Jashore the agronomic definition has relatively relaxed parameters (LWS = 3 days and AWS = 27 mm in Table 1) compared to the rainfall-based definition (50 mm over 3 days). Since the agronomic onset was developed based on the station climatology and local parameters for each station, it is more flexible and can better capture inter-annual variability of the onsets than the rainfall-based definition in Jashore. The onset dates obtained by the Dynamic 2 resulted in highest yields at the mean, median, 25^th^ and 75^th^ percentiles, and smallest standard deviation than the others, as shown in Table 2 and Fig. 5b. Statistically significant difference in simulated yields (mean, median and 75^th^ percentile) is found when the Dynamic 2 and Static 2 are compared in Table 3. In addition, the significant difference between the Dynamic 2 and Static 1 at 10^th^ percentile of the yield indicates that the Dynamic 2 can notably reduce the chances of getting yield lower (i.e., the lowest 10%) or crop failure than the Static 1.

There are two contrasting years that illustrate the potential benefit of using the Dynamic 2 agronomic definition. In 1994 and 1995, transplanting dates were found on DOY=170 and DOY= 216 (46 days of difference) respectively, which is much earlier/later than the others in 1994/1995 (Fig. 6b). In 1994, monsoon withdrew very early as indicated by the low rainfall amount in September (monthly total rainfall was 83 mm in 1994 while long-term average September monthly rainfall is 249 mm). Therefore, rice simulations with transplanting on August 8^th^ (DOY=220) by the Static 1 suffered significant water and nitrogen stress 50 days after transplanting as shown in Fig. S6, which caused crop failure in 1994 (Fig. 6c blue line). However, plants transplanted 50 days earlier on June 19^th^ (DOY = 170) by the Dynamic 2 suffered little water stress (Fig. S6b). This case shows a well-known example of late-season water stress as one of the main hazards for transplanted *aman* rice in Bangladesh (Jensen et al. 1993).

In contrast, late monsoon onset in 1995 captured by the Dynamic 2 forced a delayed transplanting on August 4^th^ (DOY=216), while Dynamic 1 induced an earlier transplanting on June 18^th^ (DOY=169; Fig. 6b). August and September rainfall in 1995 (288 and 270 mm respectively) was higher than long-term monthly average (240 and 232 mm respectively), which induced a high nitrogen leaching losses during the reproductive stage to the early transplanted rice by the Dynamic 1 due to more intense rainfall (note that N fertilizer are applied at transplanting and 25 and 60 days after transplanting). Nitrogen loss could be through leaching of nitrate converted from NH_4_^+^ when soil dries or runoff due to the weak attraction between nitrate and soil particles. However, rice transplanted later by the Dynamic 2 suffered less nitrogen stress during the reproductive stage which induced higher yield (Fig. S7a). These two contrasting cases in 1994 and 1995 suggests how important it is to adjust nursery and transplanting dates accordingly based on the inter-annually varying monsoon progression. In addition, the results show that the prediction of monsoon withdrawal (e.g., earlier or later than long-term averages or traditional conception) can be as important as the monsoon onset prediction.

#### Rajshahi

Rajshahi is in Bangladesh’s *Barind* tract and is comparatively the driest of the four study locations (Fig. 2c). It is characterized by a later monsoon onset and earlier withdrawal (Ahmed and Karmakar 1993; Montes et al. 2021b). Stiller-Reeve et al. (2015) showed that there were large differences between the average earliest and the latest onset in Rajshahi (17 pentads, ranging from April 6^th^ to June 30^th^) compared to the other stations in the south (10 or 12 pentads). The latter suggests that farmers in Rajshahi may not have a strong consensus on a local definition of monsoon onset and recognize less reliable onset date.

**Fig. 7:**
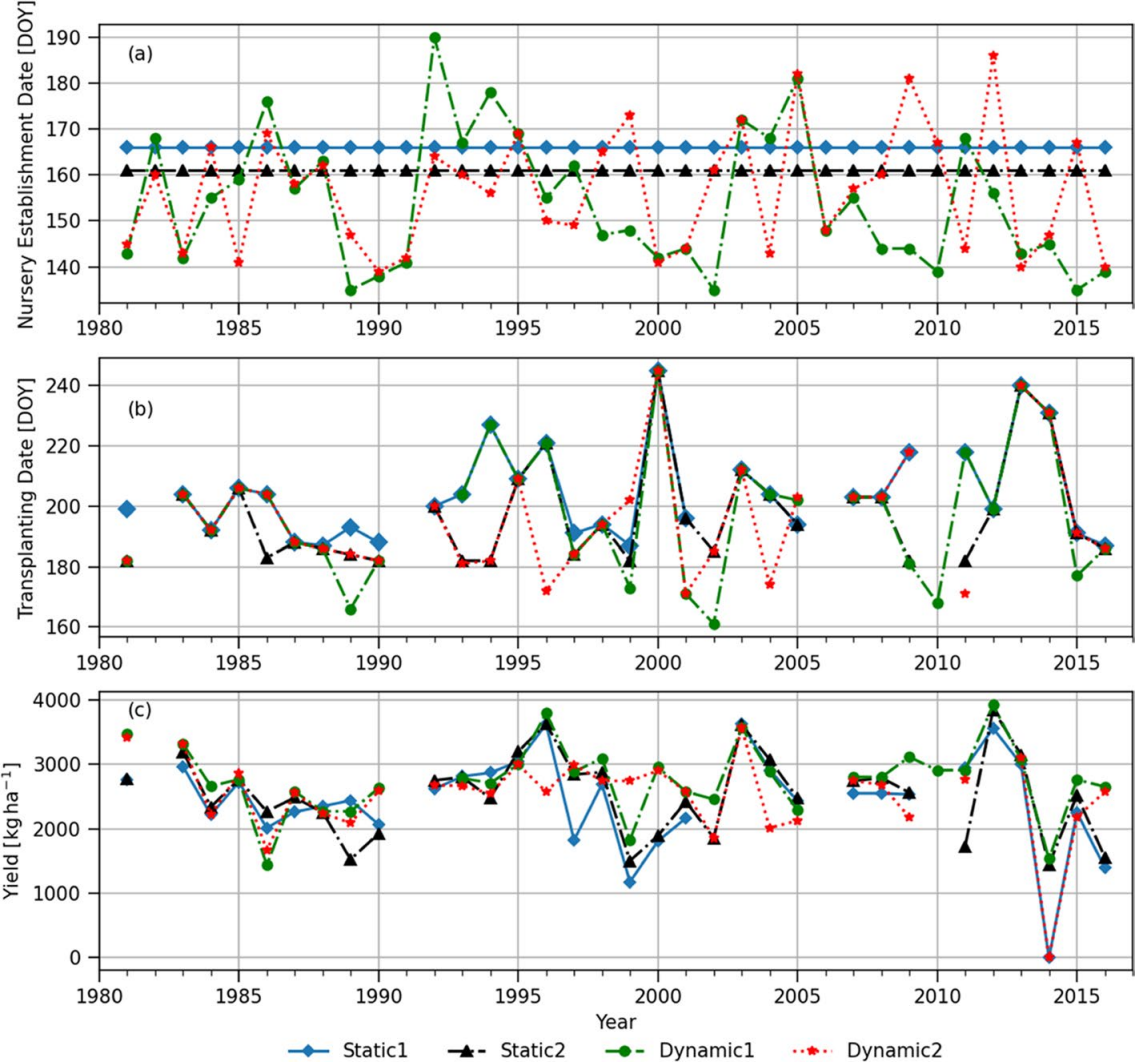
(**a**) Dates of nursery establishment corresponding to different monsoon onset definitions, (**b**) transplanting dates shown by day of year (DOY), and (**c**) simulated yields in Rajshahi. Refer to Fig. 4 for the definitions of Static 1, Dynamic 1 and Dynamic 2. Static 2 represents nursery established on June 5^th^ every year. Static2 represents nursery established on June 10^th^ every year

Dynamic onset definitions (Dynamic 1 and Dynamic 2) resulted in earlier onset dates in 70% of years (26 out of 36 years) than the traditional static onset dates (Static 1), dotted vertical lines on DOY=166 as shown in Fig. S8. The Dynamic 2 has lower values in terms of threshold numbers of both the length (3 days) and rainfall amount during the initial wet spell (31 mm) compared to the Dynamic 1. Therefore, the Dynamic 2 resulted in much earlier nursery preparation dates than the Dynamic 1 in some years (e.g., 1992, 1994, and 2004) in Fig. 7a. Due to the relatively drier conditions of Rajshahi, no transplanting dates meeting the criteria (i.e., 30 mm of ponding depth after the seedling age reaches 21 days) were found in several years (1982, 1991 and 2006), regardless of the onset definitions.

Considering yield, Dynamic 1 definition resulted in higher median yields than the others at 5% or 10% significance level (Table 3). Particularly, when compared with the Static 1, the Dynamic 1 shows statistically significant difference in 25^th^ percentile, median and mean of the yield distribution (Table 3). There are several years when the Dynamic 1 produces higher yields than the Dynamic 2. Specifically, in 2002, 2009, 2015, the Dynamic 1 takes advantage of early nursery establishment and transplanting and helps avoiding water stress during grain filling period caused by early withdrawal of monsoon season. That is, October rainfall in 2002, 2009 and 2015 was 48, 45, and 7 mm respectively while long-term average is 109 mm. The Dynamic 1 method resulted in similar or higher yields than the Dynamic 2 method except in 1999, as shown in Fig. 7c. This indicates that the agronomic onset definition parameters in Table 1, particularly six days of a post-onset dry spell could be considered as too strict to Rajshahi which has a relatively drier climate, generating late onsets and increasing risk exposure due to early withdrawal of monsoon in Rajshahi, as seen in the simulated yields of 2002, 2009 and 2015.

#### Dinajpur

**Fig. 8:**
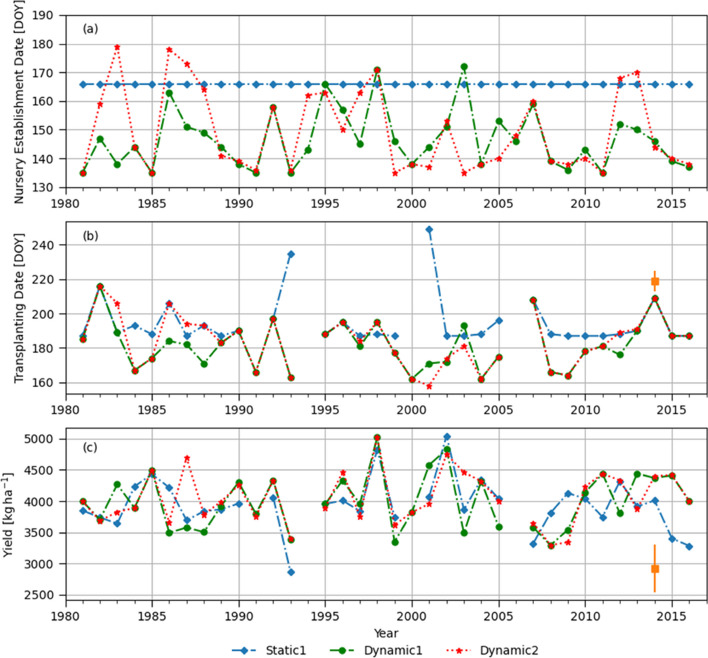
(**a**) Dates of nursery establishment corresponding to different monsoon onset definitions, (**b**) transplanting dates shown by day of year (DOY), and (**c**) simulated yields in Dinajpur. Refer to Fig. 4 for the definitions of Static 1, Dynamic 1 and Dynamic 2. Squared marks and error bars represent the mean and ± standard deviation of plot-level BIHS survey data

Dynamic onset definitions give earlier onset dates than the Static 1 in most years (Fig. 8a). 92 and 83% of the onset dates by the Dynamic 1 and Dynamic 2 definition are earlier than June 15^th^, respectively in Dinajpur (Fig. S8). Particularly, the agronomic onset definition resulted in too early onset dates having 50% of onset dates earlier than May 20^th^ (DOY=140) in Fig. S8. This indicates that the current agronomic onset definition based on the climatological average of wet or dry spells and rainfall amount allows too low thresholds causing too early onset dates in Dinajpur. Note that in the case of Dinajpur, there is no locally perceived onset dates in Stiller-Reeve et al. (2015), and thus, only one static onset date (Static 1) was used for the analysis.

Earlier nursery establishment and transplanting by the dynamic onset definitions did not lead to a significant yield increase in Dinajpur (Table 3). However, very late transplanting (August 23^rd^ DOY=253) in 1993 by the Static 1 negatively affected crop growth by exposing the rice to significant water stress during the reproductive stage (75 days after transplanting) (data not shown). In 1993, September and October rainfall (161 and 64 mm respectively) were much lower than the long-term average (270 and 102 mm respectively). This result shows another case of risk in too late transplanting due to the early demise of monsoon season.

### Sensitivity of dynamic onset definitions

In this work, we defined parameters of local agronomic onset dates based on the long-term climatology of the four locations in Bangladesh that can be considered statistically representative of the local conditions. The agronomic onset definition implies an additional level of detail but also uncertainty. For instance, the agronomic monsoon definition requires the start date from which the initial wet spell and subsequent dry spell are detected. We performed a simple sensitivity analysis to elucidate the impact of using different initial dates on the monsoon onset timing. For this, the agronomic monsoon onset was calculated using three different initial dates: May 15^th^, June 1^st^, and June 15^th^. It is observed in Table 4 that, on average, a change in one month in the initial date generates a delay of about two weeks (18 days in average) in the monsoon onset date. The earlier initial dates bring higher interannual variability (i.e., higher standard deviation) across the locations and the later initial dates allow narrower distribution of onset dates (Table 4). It is because the earlier initial date in May is likely to be within “pre-monsoon” period where interannual variability of rainfall characteristics (e.g., rainfall frequency or wet/dry spell) is high, compared to June when monsoon dominates most of the years. The higher variability in May initial date indicates the instability in pre-monsoon period and importance of taking into account dry spell through our agronomic onset definition, not to be deceived by false onset and thus to provide better guidance to farmers, not to lose benefits of early transplanting. Similar sensitivity analysis can be done for other parameters and better customized for each location, particularly Rajshahi in future studies.

**Table 4:** Averages of agronomic monsoon onset dates for three different initial dates from 1981-2017

	Day of Year			
Initial date	Barishal	Dinajpur	Jashore	Rajshahi
May 15^th^	156 (14)	150 (14)	152 (14)	157 (13)
June 1st	164 (9)	163 (8)	164 (10)	169 (10)
June 15^th^	172 (7)	172 (7)	172 (6)	174 (8)

*Values in parentheses indicates standard deviation

## Discussion

In the present study, we developed agronomic monsoon onset definitions and evaluated them against traditional practices and local farming communities' perceptions in terms of rainfed *aman* rice yield. The ultimate implication of this study is to assess the benefits of using taliroed agronomic monsoon onset definition for climate adaptation, particularly in the context of sub-seasonal to seasonal (S2S) forecasting for the rice farming community in Bangladesh. Recent advances in climate predictions, particularly at the sub-seasonal time scale, enable us to find potential for the use of the dynamic onset definitions in producing actionable agriculturally-relevant information. Increasing international collaborations has improved the understanding of multiple sources of climate predictability at scales ranging from two weeks to two months (Robertson et al. 2020; Robertson et al. 2023). There is an endeavor within the Regional Climate Outlook Forums to provide forecasts of wet- and dry-spells, and extreme rainfall frequencies (Gerlak et al. 2018), especially considering the delay in monsoon onset projected for climate change projections (Ashfaq et al. 2009). Particularly for Bangladesh, Kelley et al. (2020) found more robust predictability for wet- and dry-spells compared to total rainfalls during the monsoon season. Montes et al. (2021b) found that sea surface temperature anomalies over the Pacific Ocean (El Niño Southern Oscillation) and the Indian Ocean phases could be potential sources of predictability of onset and withdrawl of monson in Bangladesh.

The local agronomic onset definition we adopted is able to detect false onsets and thus is supposed to be more reliable than the simple rainfall-based definition, particularly in drier regions, provided its proper calibration. There are several ways to apply the estimated agronomic onset definition to produce a usable and practical “forecast” as a next step for future research. First, bias-corrected real-time daily General Circulation Models (GCMs) can be used to directly compute monsoon onsets based on the threshold numbers (Table 1). S2S forecasts or modern numerical weather prediction systems run multiple models, each starting with slightly different initial conditions, to produce ensemble daily forecasts. (Harrison et al. 2022; Pegion et al. 2019). Therefore, ensemble predictions of monsoon onset dates from the ensemble GCM outputs could be post-processed to present them in user-friendly formats (e.g., below-, near-, above-normal tercile probability categories as conventional S2S forecast or full probability distributions in probability-of-exceedance format as a flexible forecast shown in Hansen et al. 2022). Second, weather generators could be used to translate conventional and operational S2S forecasts in a probabilistic format into daily weather forecasts (e.g., Hansen and Ines 2005). Then, the generated ensemble daily weather data could be used to compute the probability of monsoon onset. Another alternative method to forecast monsoon onset is to develop a statistical model by treating the monsoon onset dates computed with historical daily weather data as a statistical predictand and GCM outputs as predictors, respectively. Hansen et al. (2006) provide more details on the above approaches, although they focus more on crop yield forecasts using seasonal climate predictions. Although there is still room to improve the dynamic onset definitions, this study takes an initial step to tailor climate information from large-scale dynamics for more agriculture-specific context in Bangladesh.

In this study, we assessed the impact of using different criteria for the date of nursery establishment and transplanting on *aman* rice yields in Bangladesh. In this sense, farmers decisions strongly depend on the seasonality in effective rainfall, and both recommended dates and those considered by farmers to establish the rice do not necessarily mean water and thermal resources to be optimally used. The assumptions and findings (e.g., advantages of earlier transplanting) of this study might not be necessarily applicable in an operational framework. For example, farmers who grow three crops a year (i.e., year-round farming) for subsistence do not necessarily have sufficient time to prepare nurseries or transplant *aman* rice after harvesting spring *aus* rice (Mahmood et al. 2004). In this case, they may have to delay transplanting in spite of the arrival of monsoon rains. Under such conditions, farmers may experience higher risks from water stress during flowering or reproductive stages. Secondly, labor is increasingly costly and challenging for farmers to access on time for optimal planting and harvesting; this can hinder farmers’ timely transplanting (Sattar 2000; Shelley et al. 2016). Furthermore, a number of other factors can constrain transplanting dates. Importantly, Barishal is located in tidal floodplains and can be greatly affected by uncontrolled floodwater during the rainy season (Shelley et al. 2016). This provides somewhat unfavorable conditions for a high-yielding variety such as BR 11. For this reason, most farmers still prefer substantially taller and hardier indigenous rice cultivars, although yield potential tends to be lower (Hamid et al., 2015) and tend to transplant very old seedlings by delaying transplanting dates.

We used the DSSAT CERES-Rice model setting up a constant soil profile and fertilizer applications at fixed rates and dates for each location, but changing weather inputs from 36 years of observations as forcing. Therefore, the model set up may not capture field-scale variability such as the heterogeneity in soil properties and varying fertilizer application rates and dates. In addition, our experiments considered only “fully rainfed” condition to focus on the impacts of inter-annual meterological varibility, excluding uncertainties from irrigation options. However, supplementary irrigation may be possible, as there are increasingly available shallow tube well and low-lift surface water irrigation systems in Bangladesh since the 1980s, which have served to increase production and de-risk rice farming (Shelley et al. 2016). Some studies (e.g., Selvaraju et al. 2006; Abdur Rashid Sarker et al. 2013) found that irrigation was the main climate change adaptation strategy of farmers, however, the significant depletion of groundwater in Bangladesh suggests this option may be less viable in the future (Kabir et al. 2017). Nonetheless, including irrigation options in crop simulations and cost-benefit analyses considering the farmers’ reluctance for irrigation due to the irrigation cost for the monsoon season *aman* rice should be assessed.

## Conclusions

The main goal of this study was to explore how different monsoon onset definitions used as criteria for crop establishment may affect rainfed *aman* season rice yields in Bangladesh. In spite of a considerable number of studies on meteorological monsoon onset and progress over Bangladesh and South Asia, literature indicates that farmers tend to have different criteria to determine the onset of the monsoon. As a consequence, farmers tend to make agricultural decisions based on experience, perception or traditional definitions. In the context of generating actionable information, this paper explored two dynamic onset definitions (rainfall threshold-based and a local agronomic definition) and analyzed rice yield response to the year-to-year varying dates for nursery establishment in comparison with the static onset definitions using the DSSAT-CERES-Rice model. Our analysis assumed fully rainfed conditions, where transplanting entirely depends on the quantity of rainfall received to achieve sufficient standing water for puddling. Dynamic onset definitions resulted in highly inter-annually varying establishment dates and in general earlier than the traditional static onset date, when applied to 36 years of weather data observed in four areas of Bangladesh. Simulation results showed that average yields slightly increased and the probability of lower yield were reduced when the monsoon onset using the dynamic onset definitions were used as a criterion for nursery establishment in relatively drier regions (Jashore and Rajshahi), while little benefit over relatively wetter regions (Barishal and Dinajpur). The benefits of the dynamic onset dates in drier regions result from the increased possibility of achieving the benefits of early transplanting by avoiding late-season abiotic stresses conditions (i.e., droughts) when the dynamic onset is used.

This exploratory study used some criteria for two dynamic onset definitions in determining nursery establishment from literature. The climatology-based local agronomic onset definition has room for further improvement by reflecting local climatic conditions (i.e., drier or wetter regimes). The crop simulations were based on certain assumptions, mainly from the literature. These assumptions may, however, need to be refined based on additional field survey data about farmers’ most common crop management practices, especially supplementary irrigation for rice transplanting. In summary, this work explored the potential value of using a refined agronomic definition of the monsoon onset aligned with the recent advances in sub-seasonal and seasonal climate forecasts as well as promising predictability of dry/wet-spells. We envision this type of locally-specific tailored forecasts would enhance the quality of agro-advisory services for farmers’ decision-making and improve the timeliness of *aman* rice nursery establishment.

### Supplementary information

ESM 1 (DOCX 1747 kb)

## Data Availability

The data that support the findings of this study are available from the corresponding author, Eunjin Han, upon reasonable request.
