# Supplementary material for: Agronomic monsoon onset definitions to support planting decisions for rainfed rice in Bangladesh
Source: Clim Change. 2024 May 13;177(5):77. doi: 10.1007/s10584-024-03736-z (PMC11090806; doi:10.1007/s10584-024-03736-z)
Supplement: Supplementary file 1 — (DOCX 1747 kb) [file 10584_2024_3736_MOESM1_ESM.docx]

**Supplementary Information**

Table S1. Genetic coefficients of the BR11 cultivar

| P1 | P2R | P5 | P2O | G1 | G2 | G3 | G4 | PHINT |
| --- | --- | --- | --- | --- | --- | --- | --- | --- |
| 825.1 | 190.8 | 337.5 | 10.7 | 44 | 0.02 | 0.8 | 0.97 | 83 |

P1 (°C-d) is the thermal time required from seedling emergence to end of the juvenile stage.

P2O (hours) is the: critical day length at which the plant development occurs at the maximum rate in photoperiod sensitivity phases.

P2R (°C-d) is the extra thermal time required when daylength is one hour longer than P2O.

PHINT (°C-d) is fixed as 83 by the model meaning that the appearance of each subsequent leaf tip requires 83 (°C-d).

P5 is the time period from beginning of grain filling to physiological maturity.

G1 is the potential spikelet number coefficient as estimated from the number of spikelets per g of main culm dry weight (less lead blades and sheaths plus spikes) at anthesis.

G2 is single grain weight (g) under ideal growing conditions.

G3 is the tillering coefficient (scaler value) relative to IR64 cultivar under ideal conditions.

G4 is the temperature tolerance coefficient.

Table S2. Soil properties of four target locations for DSSAT simulation

| Barishal  (Barishal soil series) | SLB | SLLL | SDUL | SSAT | SRGF | SSKS | SBDM | SLOC | SLCL | SLSI |
| --- | --- | --- | --- | --- | --- | --- | --- | --- | --- | --- |
|  | 5 | 0.273 | 0.425 | 0.533 | 1 | 0.12 | 1.24 | 1.74 | 47.5 | 51.4 |
|  | 7 | 0.27 | 0.434 | 0.534 | 1 | 0.12 | 1.24 | 1.65 | 47 | 52.2 |
|  | 12 | 0.281 | 0.443 | 0.536 | 1 | 0.12 | 1.22 | 1.59 | 48.4 | 51.1 |
|  | 21 | 0.331 | 0.477 | 0.56 | 1 | 0.36 | 1.17 | 2.53 | 57.2 | 39.9 |
|  | 30 | 0.261 | 0.427 | 0.534 | 0.6 | 0.09 | 1.42 | 1.56 | 45.6 | 53.6 |
|  | 37 | 0.238 | 0.404 | 0.527 | 0 | 0.09 | 1.42 | 1.53 | 42.8 | 54.8 |
|  | 51 | 0.186 | 0.366 | 0.523 | 0 | 0.15 | 1.46 | 1.57 | 33.7 | 66.2 |
|  | 67 | 0.24 | 0.406 | 0.529 | 0 | 0.09 | 1.42 | 1.54 | 42.9 | 55 |

| Jashore  (Gangni soil series) | SLB | SLLL | SDUL | SSAT | SRGF | SSKS | SBDM | SLOC | SLCL | SLSI |
| --- | --- | --- | --- | --- | --- | --- | --- | --- | --- | --- |
|  | 12 | 0.29 | 0.43 | 0.53 | 1 | 0.09 | 1.38 | 1.46 | 51 | 40 |
|  | 19 | 0.32 | 0.44 | 0.52 | 0.5 | 0.06 | 1.45 | 0.89 | 55 | 39 |
|  | 36 | 0.31 | 0.44 | 0.54 | 0.35 | 0.09 | 1.41 | 0.45 | 53 | 40 |
|  | 52 | 0.26 | 0.41 | 0.5 | 0 | 0.09 | 1.44 | 0.32 | 44 | 53 |
|  | 70 | 0.134 | 0.377 | 0.467 | 0 | 0.15 | 1.38 | 0.22 | 32 | 66 |
|  | 89 | 0.154 | 0.347 | 0.437 | 0 | 0.68 | 1.36 | 0.13 | 25 | 69 |
|  | 110 | 0.068 | 0.243 | 0.337 | 0 | 0.68 | 1.2 | 0.1 | 10 | 66 |
|  | 130 | 0.061 | 0.24 | 0.334 | 0 | 0.68 | 1.02 | 0.1 | 10 | 63 |

| Rajshahi  (Ishurdi series) | SLB | SLLL | SDUL | SSAT | SRGF | SSKS | SBDM | SLOC | SLCL | SLSI |
| --- | --- | --- | --- | --- | --- | --- | --- | --- | --- | --- |
|  | 18 | 0.224 | 0.396 | 0.449 | 1 | 0.15 | 1.39 | 1.24 | 39.8 | 54.4 |
|  | 36 | 0.235 | 0.407 | 0.434 | 0.583 | 0.09 | 1.43 | 0.85 | 41.5 | 57.1 |
|  | 62 | 0.277 | 0.341 | 0.438 | 0.375 | 0.09 | 1.42 | 0.75 | 47.7 | 52 |
|  | 91 | 0.321 | 0.376 | 0.442 | 0 | 0.09 | 1.41 | 0.45 | 54.2 | 45 |

| Dinajpur  (Ruhea series) | SLB | SLLL | SDUL | SSAT | SRGF | SSKS | SBDM | SLOC | SLCL | SLSI |
| --- | --- | --- | --- | --- | --- | --- | --- | --- | --- | --- |
|  | 18 | 0.137 | 0.256 | 0.447 | 1 | 2.59 | 1.37 | 1.51 | 13.9 | 21.6 |
|  | 57 | 0.154 | 0.277 | 0.453 | 0.472 | 2.59 | 1.35 | 1.77 | 15.9 | 19.5 |
|  | 70 | 0.096 | 0.175 | 0.4 | 0.281 | 2.59 | 1.52 | 0.56 | 11 | 13.5 |
|  | 79 | 0.054 | 0.107 | 0.409 | 0.225 | 0.06 | 1.5 | 0.36 | 3.6 | 5.4 |
|  | 89 | 0.045 | 0.088 | 0.403 | 0.186 | 0.06 | 1.52 | 0.2 | 2.8 | 2 |

SLB Depth, base of layer, cm

SLLL Lower limit, cm3 cm^-3^

SDUL Upper limit, drained, cm3 cm^-3^

SSAT Upper limit, saturated, cm3 cm^-3^

SRGF Root growth factor, soil only, 0.0 to 1.0

SSKS Saturated hydraulic conductivity, cm h-^1^

SBDM Bulk density, moist, g cm^-3^

SLOC Organic carbon, %

SLCL Clay (<0.002 mm), %

SLSI Silt (0.05 to 0.002 mm), %

Table S3. Summary statistic of 2014 crop management and yield data taken from the Bangladesh Integrated Household Survey (BIHS) 2015

|  |  | **Transplanting date (DOY)** | | | **Yields [Kg ha-1 ]** | | |
| --- | --- | --- | --- | --- | --- | --- | --- |
|  |  | Mean | STDEV | No. of Data | Mean | STDEV | No. of Data |
| Rainfed | Barisal | 219.05 | 18.94 | 178 | 2980 | 1160 | 178 |
|  | Jessore | 204.48 | 18.87 | 156 | 3270 | 1090 | 156 |
|  | Rajshahi | N/A | N/A | N/A | N/A | N/A | N/A |
|  | Dinajpur | 218.75 | 5.80 | 4 | 2920 | 380 | 4 |
| Irrigated | Barisal | 202.72 | 16.92 | 32 | 3040 | 1040 | 32 |
|  | Jessore | 203.84 | 14.79 | 133 | 4020 | 1750 | 133 |
|  | Rajshahi | 212.51 | 10.75 | 78 | 5780 | 1190 | 78 |
|  | Dinajpur | 199.93 | 8.72 | 219 | 5170 | 1480 | 219 |

*Note: The raw data can be found in (International Food Policy Research, 2016; Sapkota et al., 2021). The table shows summary of only transplanting dates and yields from rainfed farms (i.e., irrigated field data were excluded) in the selected divisions of interest. Fig. S5 shows the locations of the field survey was conducted.

Table S4. An example of DSSAT CERES Rice experiment file (RIX)

| 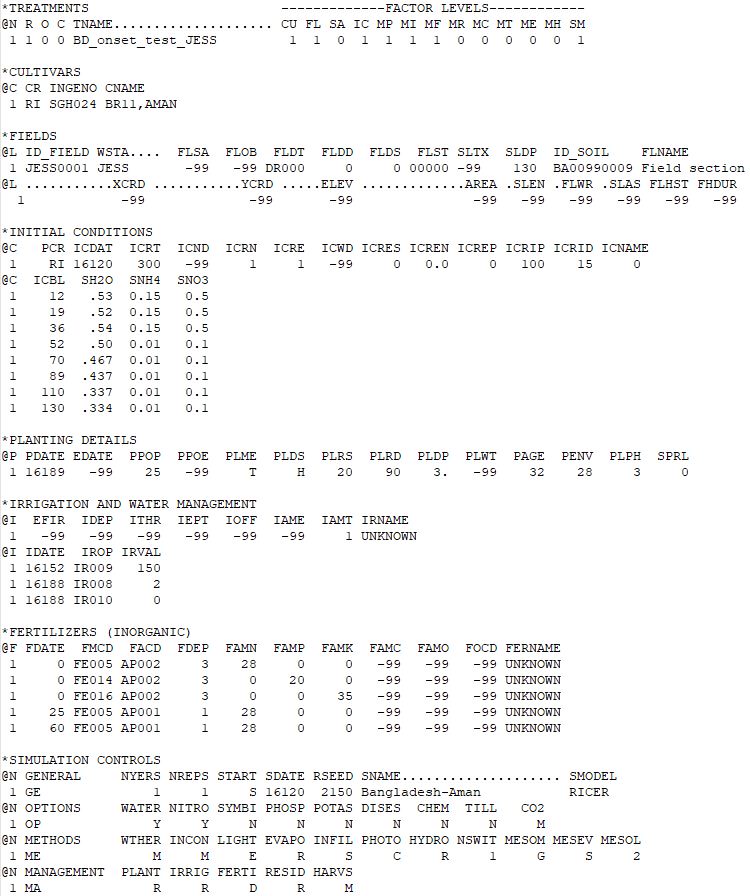 |
| --- |

**
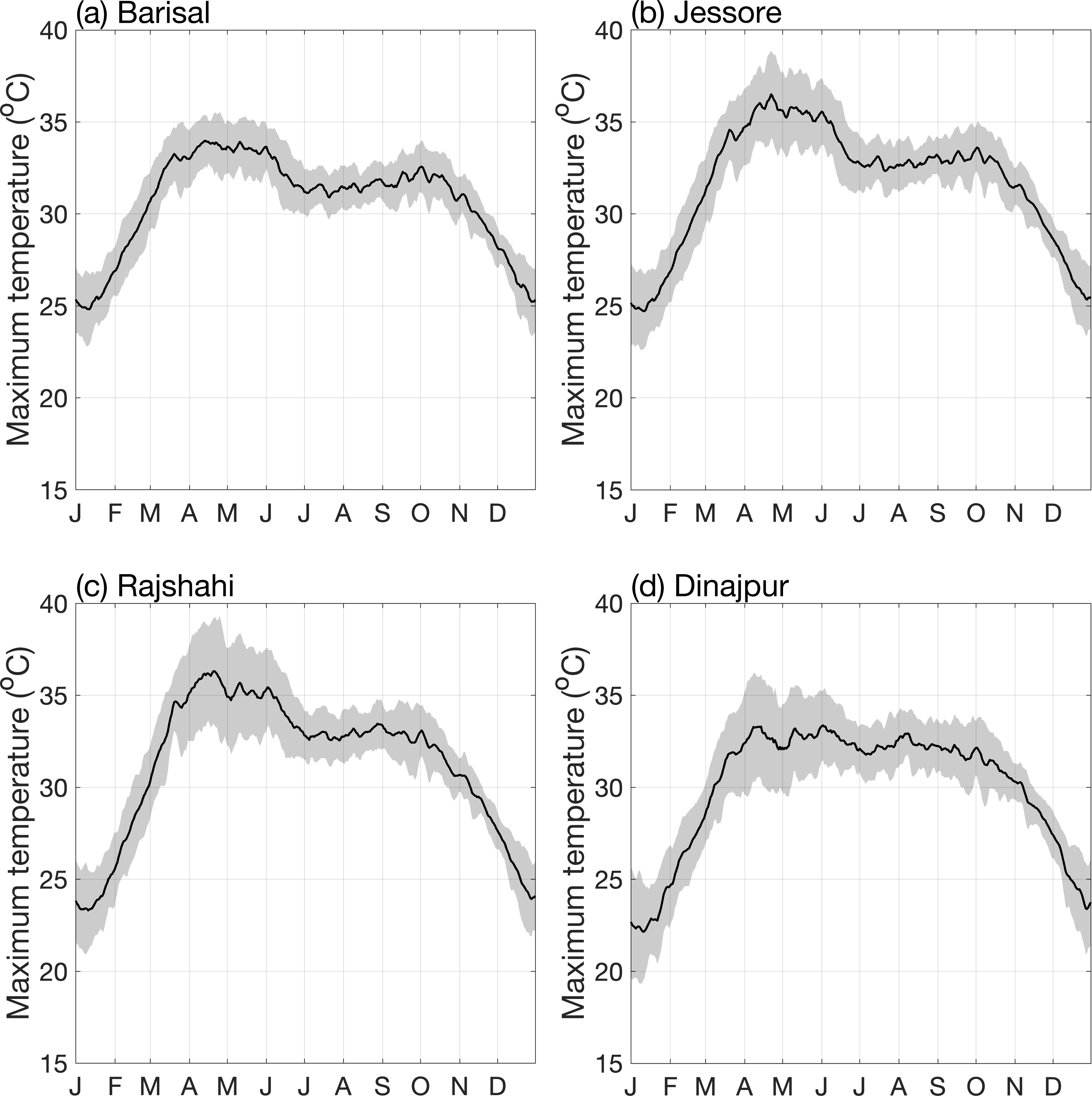
**

Fig. S1. Mean annual cycle (1981-2017) of the 5-day moving average of maximum temperature for the four locations in Bangladesh. The shaded area corresponds to the interannual standard deviation.

**
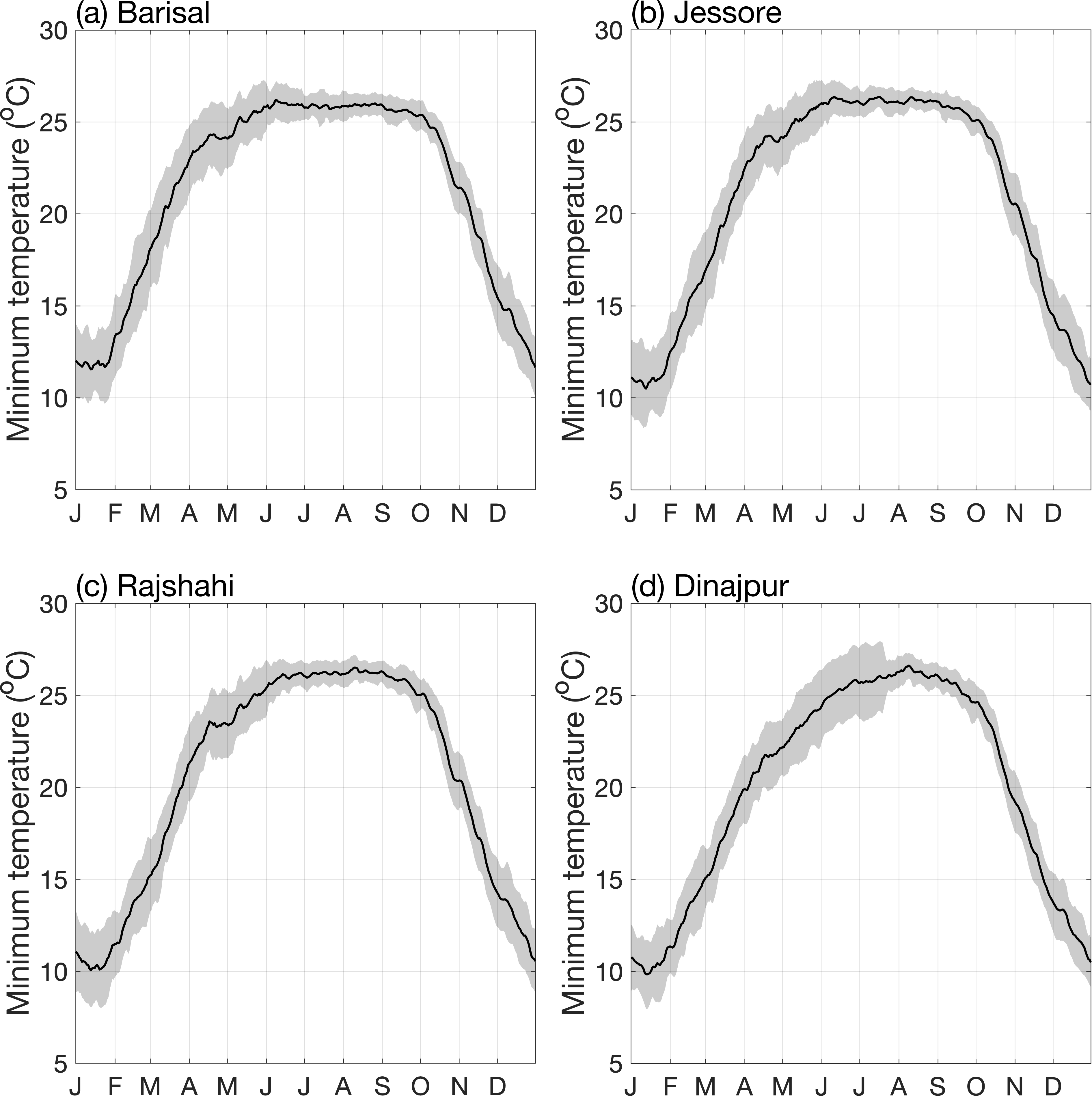
**

Fig. S2. Mean annual cycle (1981-2017) of the 5-day moving average of minimum temperature for the four locations in Bangladesh. The shaded area corresponds to the interannual standard deviation.

**
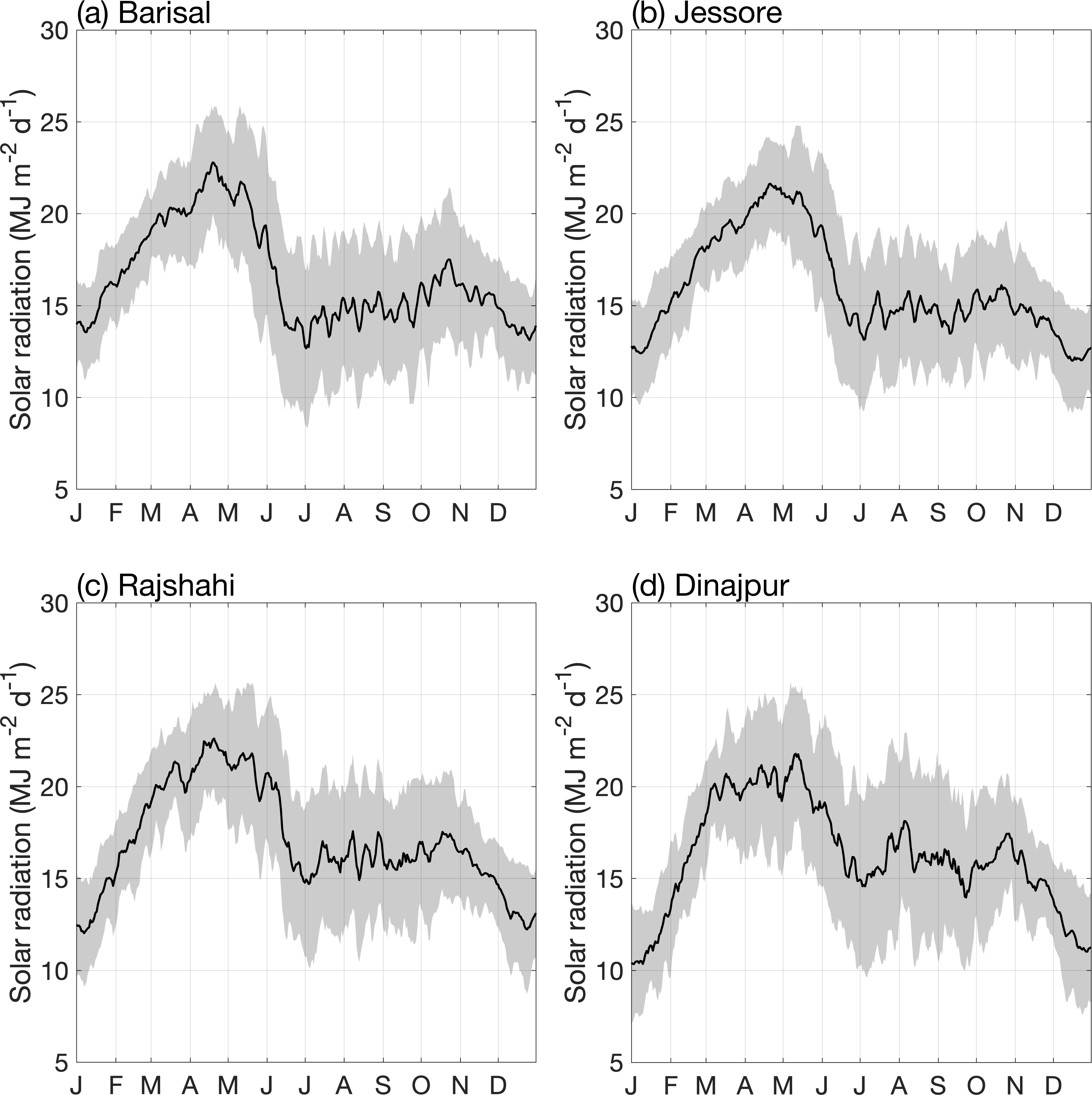
**

Fig. S3. Mean annual cycle (1981-2017) of the 5-day moving average of solar radiation for the four locations in Bangladesh. The shaded area corresponds to the interannual standard deviation.

**
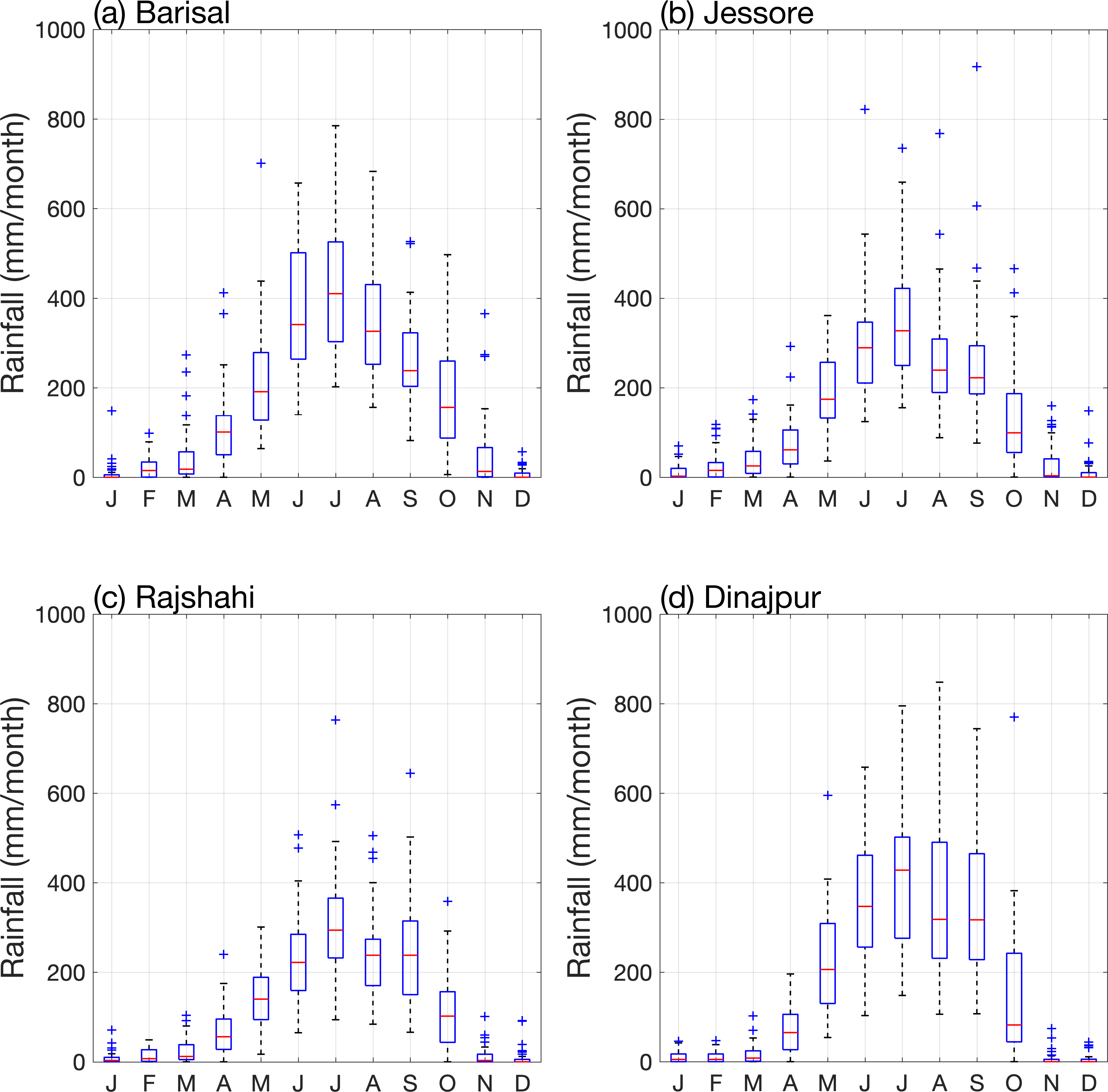
**

Fig. S4. Boxplots of annual distribution of monthly rainfall for the four locations in Bangladesh. Central mark shows the median and the edges are the 25^th^ and 75^th^ percentiles; dashed lines extend to the most extreme values not considered outliers, and outliers are plotted individually (plus sign).

**
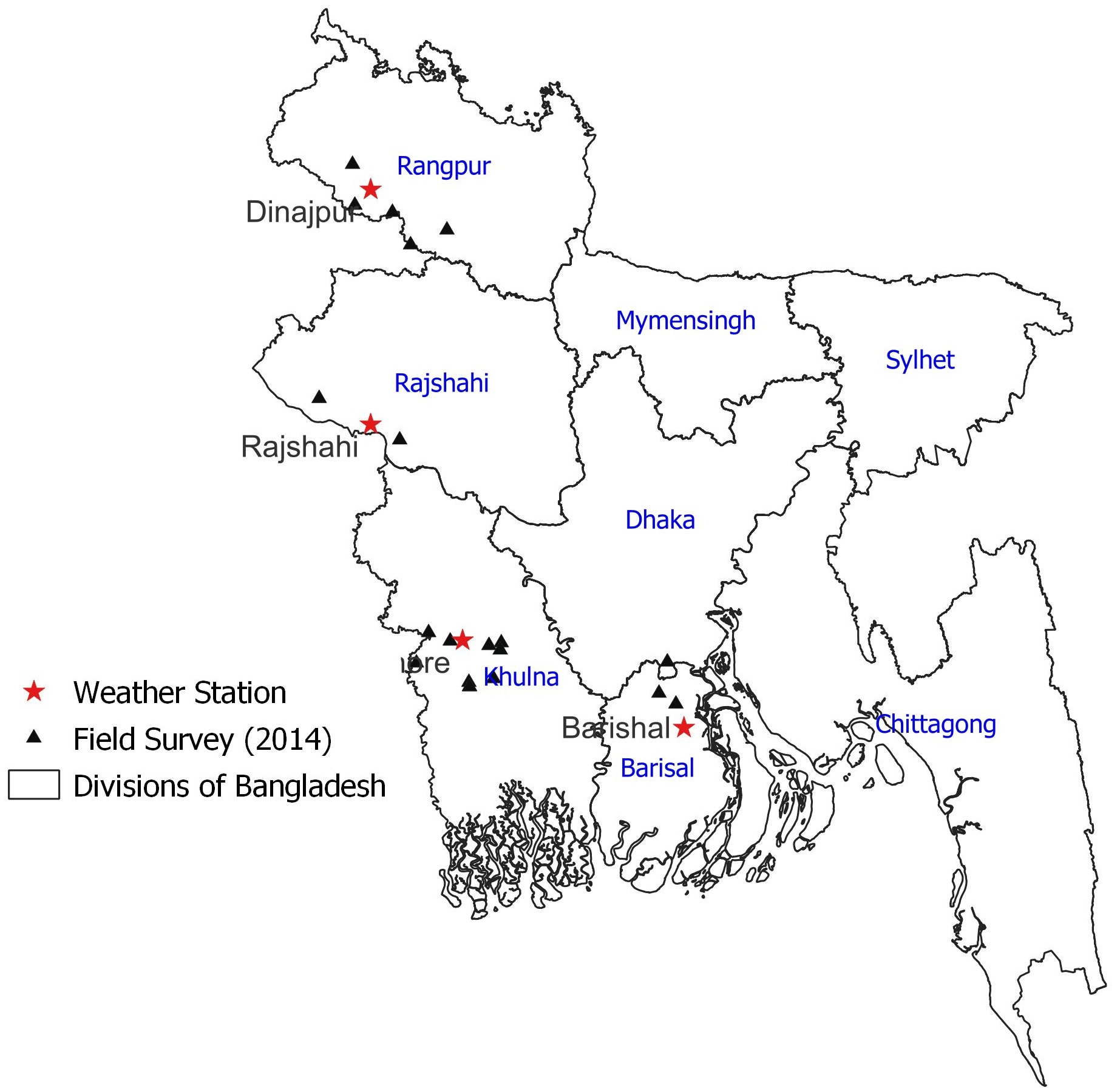
**

**Fig. S5.** Selected locations of field survey conducted in 2014 and used for validating simulation results of DSSAT CERES Rice model.


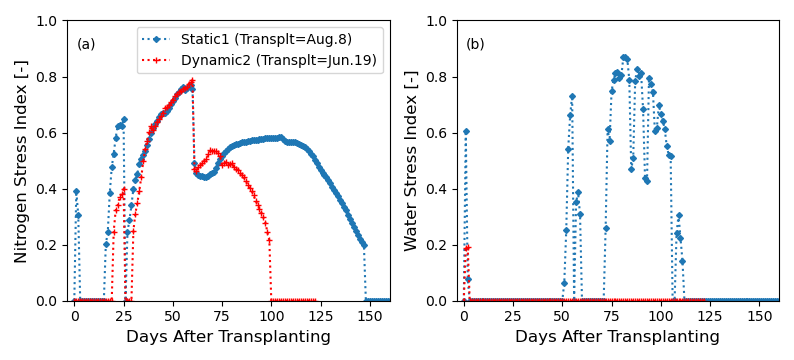


**Fig. S6.** Comparison of (a) nitrogen and (b) water stress indices between the simulation results of the Static 1 and Dyanmic 2 definitions in 1994 in Jashore. Note that the nitrogen (or water) stress index is computed as 1 minus nitrogen (or water) deficiency, where nitrogen (or water) deficiency is the ratio of supply to potential demand. Refer to Ritchie (1998) and Godwin and Singh (1998) for more details on the plant nitrogen (or water) stress estimation.


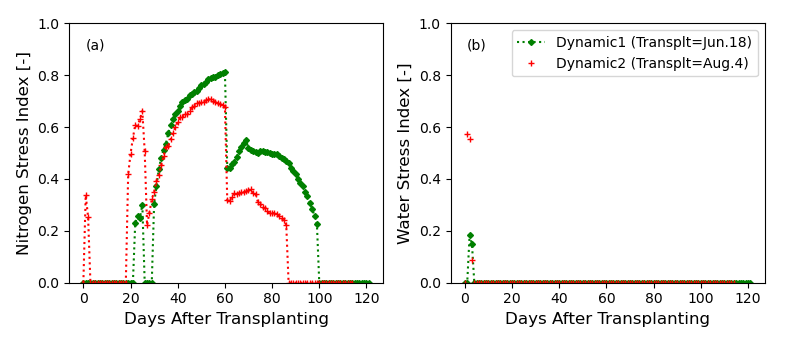


**Fig. S7.** Comparison of (a) nitrogen and (b) water stress indices between the simulation results of Dynamic 1 and Dyanmic 2 definitions in 1995 in Jashore.


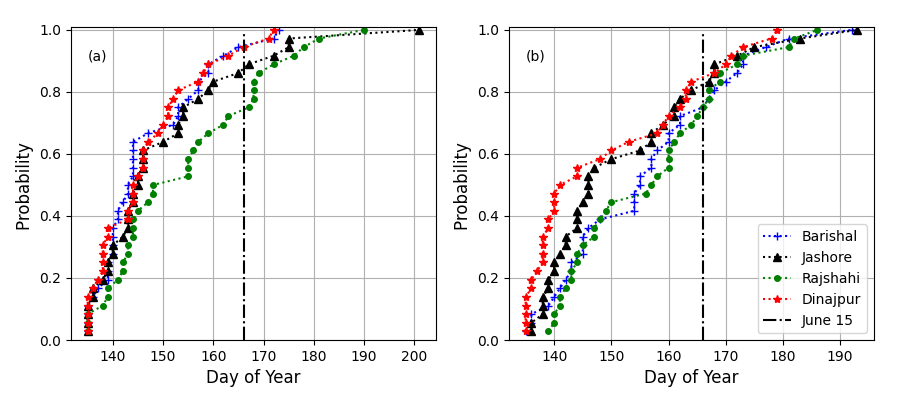


**Fig. S8.** Cumulative distribution of estimated onset dates for each location by (a) the rainfall-based (Dynamic 1) and (b) agronomic (Dynamic 2) onset definitions.

Reference

Godwin, D. and Singh, U., 1998. Nitrogen balance and crop response to nitrogen in upland and lowland cropping systems, Understanding options for agricultural production. Springer, pp. 55-77.

International Food Policy Research, I., 2016. Bangladesh Integrated Household Survey (BIHS) 2015. In: I.F.P.R. Institue (Editor). Household- and Community-level Surveys. <https://doi.org/10.7910/DVN/BXSYEL>, Harvard Dataverse.

Ritchie, J.T., 1998. Soil water balance and plant water stress, Understanding options for agricultural production. Springer, pp. 41-54.

Sapkota, T.B. et al., 2021. Spatially explicit database on crop-livestock management, soil, climate, greenhouse gas emissions and mitigation potential for all of Bangladesh. Data in brief, 37: 107225.
